# Synthesis, Crystal Structures, and Spectroscopic Properties of Novel Gadolinium and Erbium Triphenylsiloxide Coordination Entities

**DOI:** 10.3390/molecules27010147

**Published:** 2021-12-27

**Authors:** Patrycja Wytrych, Józef Utko, Julia Kłak, Maciej Ptak, Mariusz Stefanski, Tadeusz Lis, Jolanta Ejfler, Łukasz John

**Affiliations:** 1 Faculty of Chemistry, University of Wrocław, 14 F. Joliot-Curie, 50-383 Wrocław, Poland; patrycja.wytrych@chem.uni.wroc.pl (P.W.); jozef.utko@chem.uni.wroc.pl (J.U.); julia.klak@chem.uni.wroc.pl (J.K.); tadeusz.lis@chem.uni.wroc.pl (T.L.); jolanta.ejfler@chem.uni.wroc.pl (J.E.); 2 Institute of Low Temperature and Structure Research, Polish Academy of Sciences, 2 Okólna, 50-422 Wrocław, Poland; m.ptak@intibs.pl (M.P.); m.stefanski@intibs.pl (M.S.)

**Keywords:** lanthanide coordination chemistry, triphenylsilanol, triphenylsiloxide, gadolinium triphenylsiloxide, erbium triphenylsiloxide, X-ray crystallography, IR spectroscopy, Raman spectroscopy, absorption spectroscopy, magnetism

## Abstract

In alkali metal and lanthanide coordination chemistry, triphenylsiloxides seem to be unduly underappreciated ligands. This is as surprising as that such substituents play a crucial role, among others, in stabilizing rare oxidation states of lanthanide ions, taking a part of intramolecular and molecular interactions stabilizing metal-oxygen cores and many others. This paper reports the synthesis and characterization of new lithium [Li_4_(OSiPh_3_)_4_(THF)_2_] (**1**), and sodium [Na_4_(OSiPh_3_)_4_] (**2**) species, which were later used in obtaining novel gadolinium [Gd(OSiPh_3_)_3_(THF)_3_]·THF (**3**), and erbium [Er(OSiPh_3_)_3_(THF)_3_]·THF (**4**) configuration, it can result in res were determined for all **1**–**4** compounds, and in addition, IR, Raman, absorption spectroscopy studies were conducted for **3** and **4** lanthanide compounds. Furthermore, direct current (dc) variable-temperature magnetic susceptibility measurements on polycrystalline samples of **3** and **4** were carried out in the temperature range 1.8–300 K. The **3** shows behavior characteristics for the paramagnetism of the Gd^3+^ ion. In contrast, the magnetic properties of **4** are dominated by the crystal field effect on the Er^3+^ ion, masking the magnetic interaction between magnetic centers of neighboring molecules.

## 1. Introduction

In scientific literature, metal siloxides are often associated with metal alkoxides, although this affinity shows noticeable differences in unique structures than related alkoxides and aryloxides [1]. Simultaneously, it is worth noting that the coordination chemistry of the organosiloxide analogs is not well understood and is widely described. The decreased basicity clearly distinguishes triorganosiloxides from alkoxide derivatives, significantly reducing the possibility of bridging connections between metal centers. Consequently, the triorganosiloxides may experience a difference in the degree of covalency compared with alkoxide analogs. For instance, the metal tetrasiloxides differ from the metal alkoxides in their degree of aggregation. This phenomenon is responsible for the more negligible steric effect of trialkylsiloxo compared with tertiary alkoxo substituents.

On the other hand, due to the absence of an energetically accessible vacant *d*_π_ manifold, they can be used to synthesize homo- and heterometallic coordination entities [2,3], leading to, among others, efficient catalysts [4] and molecular precursors for solid-state metal silicate-based materials [5]. Such molecular precursors guarantee dispersion of metal sources at the molecular level and provide uniform reaction sites for conversion to the ceramic. Moreover, metal triorganosiloxides, compared to metal alkoxides, are less labile to hydrolysis, which may be ascribed to the water-repelling property of triorganosiloxide groups.

The first examples of the alkali metal salts of silanols and their application in coordination chemistry were reported in the middle of the twentieth century [6]. Among this group of silicon compounds, a special place belongs to triphenylsiloxides. This interest derives from the fact that the presence of phenyl rings affects the shape of the individual unit of the species and the entire crystal structure. Moreover, their occurrence influences intramolecular and molecular interactions between the aryl molecule fragments and alkali metal cations. Surprisingly, the number of triphenylsilanol/triphenylsiloxide structures described in the literature is small. For instance, Caulton et al. described synthesis and structures of lithium and potassium triphenylsiloxides of formulae [Li(OSiPh_3_)(DME)]_2_, [K_4_(OSiPh_3_)_4_(DME)]_2_ (where DME = dimethoxyethane), and [K(18-crown-6)(OSiPh_3_)]_2_ [7]. There are also known other lithium [Li_4_(OSiPh_3_)_2_(THF)_4_]Cl_2_ [8] and [Li_2_(OSiPh_3_)_2_(DMPU)_2_] [9] (where DMPU = N,N′-dimethylpropyleneurea), and sodium [Na_4_(OSiPh_3_)_4_(H_2_O)_3_] [10] species. Our group also reported two potassium triphenylsiloxides [K_6_(OSiPh_3_)_6_(H_2_O)(C_3_H_7_OH)]·2C_6_H_5_CH_3_ and [K_6_(OSiPh_3_)_6_(H_2_O)_2_] showing polyoxometalate-like architectures [11]. Metal triphenylsiloxides can be used for other modifications, e.g., they can react with metal alkoxides, oxo-alkoxides, amides to form the corresponding homo- and heteroleptic metal siloxides [12,13].

Like alkali metal species, the group of lanthanide complexes supported by triorganosiloxide ligands is insignificant. However, siloxides as substituents play a crucial role. A great example of this is the stabilizing effect of the oxidation state +4 in synthesizing lanthanides species other than cerium(IV). In this regard, Mazzanti et al. reported on the monodentatetriphenylsiloxide ligand and its role in the isolation of a molecular [Tb(OSiPh_3_)_4_(MeCN)_2_] compound despite its inability to saturate the coordination sphere of the metal center [14]. Oddly, there are few examples of compounds’ structures in the literature in which monodentatetriphenylsiloxide ligands surround the lanthanide ions. The first lanthanide complexes containing triorganosiloxide anions were described at the end of the twentieth century. For example, Evans et al. reported the synthesis of [Ce(OSiPh_3_)_3_(THF)_3_](THF) starting from “NaOSiPh_3_” and anhydrous cerium nitrate [15]. In that article, the authors emphasized that cerium nitrates can be alternatively used instead of oxo-alkoxides which are also commonly applied substrates. Thus, the conversion of oxo-alkoxide precursor led to oxide-free triorganosiloxides. Another example was outlined by Caulton et al., who reported on lanthanum triphenylsiloxide, which was obtained in the reaction of [La{N(SiMe_3_)_2_}_3_] with Ph_3_SiOH in toluene, giving high yields of [La(OSiPh_3_)_3_(THF)_3_] [16]. These two synthetic strategies seemed simple, but the papers dealt only with the X-ray crystal structures, and no other physicochemical analyses were performed. Thus, up to now, homometallictriorganosiloxide chemistry remains poorly developed.

This paper reports the syntheses of lithium and sodium triphenylsiloxides and their use as transmetalation reagents for high yield obtaining gadolinium and erbium derivatives, which are the project’s primary goals. It is common knowledge that the coordination chemistry of lanthanide(III) ions has been extensively studied in recent years because the resulting complexes can be conveniently employed as useful devices or probes in various fields. Lanthanide ions and their complexes are of particular interest in molecular magnetism due to their large and anisotropic magnetic moment. Furthermore, they are involved in molecular architectures that exhibit single-molecule magnet (SMM), single-chain magnet (SCM), and single-ion magnet (SIM) behaviors with fascinating potentialities in terms of magnetic anisotropy [17,18,19]. The large magnetic moment and spin-lattice relaxation associated with the Gd^3+^ ion have been used for various applications, such as magnetic resonance imaging (MRI) [20], and to develop molecular-based magnetic materials [21] and molecular coolants [22]. Additionally, mononuclear Gd^3+^ complexes have been used as an alternative for spin-labeling experiments to study the structural dynamics and conformational changes in biological molecules and biomacromolecules [23].

## 2. Experimental

### 2.1. Materials and Methods

Ethanol (HPLC grade, J.T. Baker, Gdańsk, Poland), toluene (HPLC grade, J.T. Baker, Gdańsk, Poland), tetrahydrofuran (Chempur), hexane (HPLC grade, J.T. Baker, Gdańsk, Poland), DCM (HPLC grade, J.T. Baker). All solvents, except hexane, were used with further purification. Toluene and tetrahydrofuran were purified by distillation with metallic sodium, and ethanol was purified by distillation with metallic magnesium. Triphenylsilanol (98%), gadolinium(III) chloride anhydrous (99.99%), erbium(III) chloride anhydrous (99.99%), metallic lithium (99%), metallic sodium (99.9%) were purchased from Sigma Aldrich (Darmstadt, Germany) and used without further purification.

FT-IR spectra of **1** and **2** crystals were measured using a Bruker Vertex 70 FTIR spectrometer (Ettlingen, Germany) in the transmission mode. Sample spectra were recorded in Nujol mulls sandwiched between CsI plater. The FTIR sample chamber was flushed continuously with N_2_ prior to data acquisition in the range 4000−400 cm^−1^ with a precision of ±1 cm^−1^.

The IR spectra of **3**, **4**, and the Ph_3_SiOH substrate were measured using a Nicolet iS50 spectrometer (Waltham, MA, USA) equipped with a diamond ATR (attenuated total reflection) accessory. The ATR correction was used to process spectra analysis. The transmittance IR spectrum of liquid THF was recorded between two CsI crystals. The spectral resolution was set to 2 cm^−1^.

Raman spectra of the **3** and **4** crystals, Ph_3_SiOH, and liquid THF were measured using a Bruker MultiRAM spectrometer (Billerica, MA, USA) with a YAG:Nd laser excitation (1064 nm).

Elemental analyses (C, H) were performed using a Vario EL III element analyzer (Hanau, Germany). Quantitative analyses of Na, Li, Gd, and Er were carried out using emission spectrometer iCAP 7400 DUO icp - Thermo Fisher Scientific (Waltham, MA, USA).

The diffuse reflectance measurements of polycrystalline **3**, **4,** and Ph_3_SiOH were performed in the back-scattering mode using an Agilent Cary 5000 spectrophotometer (Santa Clara, CA, USA). The Al_2_O_3_ powder served as a reference.

Magnetic susceptibility and magnetization measurements were carried out on a Quantum Design SQUID magnetometer (type MPMS-XL) (Darmstadt, Germany). Direct current (dc) magnetic measurements were performed on polycrystalline samples of **3** and **4** in the 1.8–300 K temperature range with an applied external magnetic field of 0.5 T. Diamagnetic corrections estimated with Pascal constants were applied [24]. To prevent torqueing, the samples were restrained in a drop of paraffin oil. 

Diffraction data for all crystals were collected at 100 K on monocrystalline diffractometers with CCD cameras. The CrysAlisPro software from Oxford Diffraction was used to determine cell parameters, data reduction, and absorption correction for all crystals. The structures were solved by direct methods (SHELXS97) and refined using the least-squares technique (SHELXL2013). CCDC Nos.: 2088719 (**1**), 2088720 (**2**), 2088717 (**3**), and 2088718 (**4**).

### 2.2. Syntheses

All reactions and operations were performed under an inert atmosphere of N_2_ using standard Schlenk apparatus and vacuum line techniques. Lithium and sodium ethoxides were obtained using a slightly modified recipe reported in the literature [25]. Metallic lithium/sodium was mixed with ethanol and toluene under an inert atmosphere at room temperature.

#### 2.2.1. Synthesis of [Li_4_(OSiPh_3_)_4_(THF)_2_] (**1**)

To a stirred on an ice bath solution of lithium ethoxide (0.624 g, 1.2 mmol) in THF (40 mL), the solution of Ph_3_SiOH (3.314 g, 1.2 mmol) in THF (40 mL) was added dropwise. The obtained mixture was stirred for 3 h, after which it was warmed to room temperature and evaporated to dryness. The residue was dissolved in THF (10 mL), and the mixture was heated under reflux until a clear solution was visible. The obtained clear solution was cooled to room temperature and left until a colorless crystal of **1** appeared after some days as the main product. Then, it was filtered off and dried in vacuo. Yield: 76% (2.57 g, 2.02 mmol). FT-IR (cm^−1^, nujol): *ν*_C-H_ = 3062 (m), γ_C-H_ = 1462 (s), ν_C-C + C-H_ = 1426 (s), ν_C-H_ = 1377 (m), δ_Si-O_ = 1113 (m), γ_C-H+_ν_C=O_ = 979 (s), γ_C-H+_δ_C=O+_γ_C-H_ = 703 (s), δ_O-Si-C_ = 530 (m). Elemental analyses calcd (%) for C_80_H_76_Li_4_O_6_Si_4_: C 75.45; H 6.01; Li 2.18; found C 75.05; H 5.93; Li 2.03.

#### 2.2.2. Synthesis of [Na_4_(OSiPh_3_)_4_] (**2**)

To a stirred solution of sodium ethoxide (0.945 g, 13.9 mmol) in THF (20 mL), the solution of Ph_3_SiOH (4.06 g, 13.9 mmol) in THF (20 mL) was added dropwise and stirred for 24 h at room temperature. Then, the solution was evaporated to dryness, and the residue was dissolved in 10 mL of THF. The obtained clear solution was left at room temperature until a colorless crystals of **2** appeared after some days. Finally, the product was filtered off and dried in vacuo. Yield: 89% (3.68 g, 3.08 mmol). FT-IR (cm^−1^, nujol): ν_C-H_ = 3063 (m), γ_C-H_ = 1462 (s), ν_C-C+C-H_ = 1427 (s), ν_C-H_ = 1377 (m), δ_Si-O_ = 1110 (m), γ_C-H+_ν_C=O_ = 995 (m), γ_C-H+_δ_C=O+_γ_C-H_ = 702 (s), δ_O-Si-C_ = 521 (s). Elemental analyses calcd (%) for C_72_H_60_Na_4_O_4_Si_4_: C 72.45; H 5.07; Na 7.70; found C 72.05; H 5.13; Na 7.32.

#### 2.2.3. Synthesis of [Gd(OSiPh_3_)_3_(THF)_3_]·THF (**3**)

The compound **1** (2.0351 g, 7.206 mmol) and anhydrous gadolinium chloride (0.4751 g, 1.8 mmol) were dissolved in 70 mL of THF and stirred for 24 h at room temperature. Then, the solution was evaporated to dryness, the residue was treated by DCM, and side product precipitate appeared, which was filtered off. Next, the filtrate was evaporated, and the residue was dissolved in THF. The obtained clear solution was left at room temperature until colorless crystal appeared after four days as main product **3**, which was filtered off and dried in vacuo. Note: Instead of **1**, sodium triphenylsiloxide **2** can be used. The synthetic protocol is the same as applied for **1**. Yield: 47% (1.27 g, 0.998 mmol). Elemental analyses calcd (%) for C_70_H_77_GdO_7_Si_3_: C 66.10; H 6.10; Gd 12.06; found C 65.98; H 6.02; Gd 11.88.

#### 2.2.4. Synthesis of [Er(OSiPh_3_)_3_(THF)_3_]·THF (**4**)

The compound **2** (1.4764 g, 4.82 mmol) and anhydrous erbium chloride (0.8450 g, 1.61 mmol) were dissolved in a mixture of THF (20 mL) and toluene (40 mL) and stirred for 24 h at room temperature. Then, the solution was evaporated to dryness, the residue was treated by DCM, and side product precipitate appeared, which was filtered off. Next, the filtrate was evaporated, and the residue was dissolved in THF. The obtained clear solution was left at room temperature until pale pink crystals appeared after one week as the main product **4**. Note: Instead of **2**, **1** can be used. The synthetic protocol is the same as applied with sodium triphenylsiloxide **2**. Yield: 75% (1.232 g, 0.961 mmol). Elemental analyses calcd (%) for C_70_H_77_ErO_7_Si_3_: C 65.59; H 6.05; Er 13.05; found C 65.23; H 5.98; Er 12.64.

## 3. Results and Discussion

### 3.1. Syntheses and Molecular Structures of **1**–**4**

**Scheme 1:**
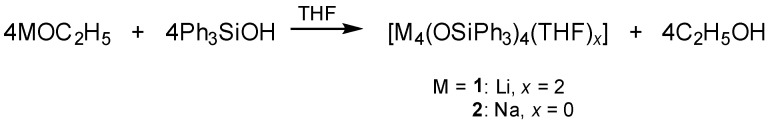
Synthesis reaction scheme of **1** and **2**.

In order to obtain erbium and gadolinium triphenylsiloxides, in the first step, the starting lithium [Li_4_(OSiPh_3_)_4_(THF)_2_] (**1**) and sodium [Na_4_(OSiPh_3_)_4_] (**2**) derivatives were synthesized as shown in Scheme 1, and both compounds were obtained in a crystalline form. 

The formation of **1** and **2** was confirmed by elemental analysis and infrared spectroscopy. Unfortunately, because of the dynamic behavior of the resulting species in the solution, we were not able to interpret NMR spectra. Nevertheless, as these two compounds were obtained in a crystalline form, their crystal structures were determined.

**Figure 1:**
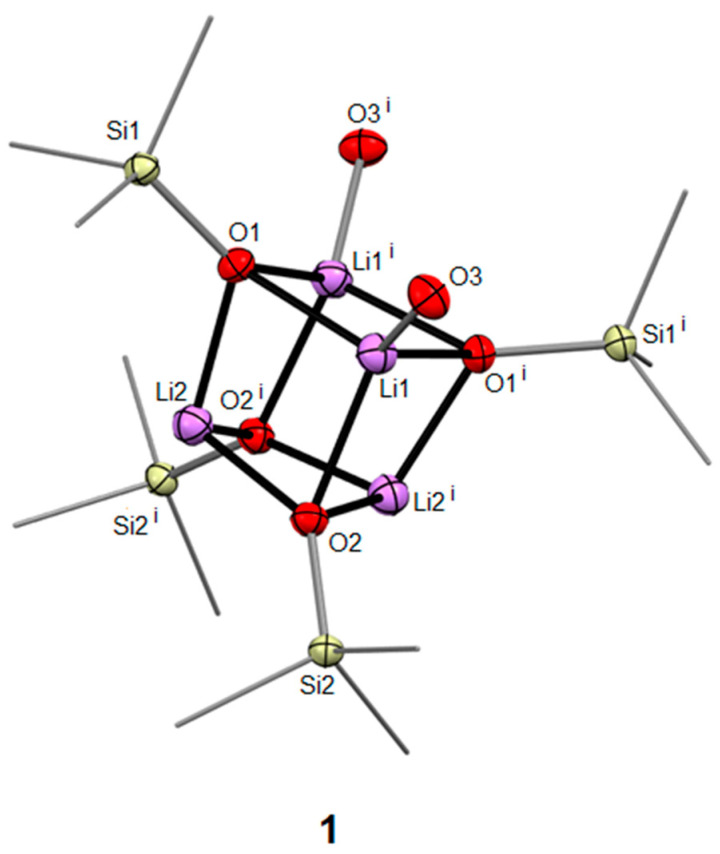
Molecular structure of **1** (50% probability ellipsoids). Hydrogen atoms and phenyl and THF groups are omitted for clarity. Symmetry code: (i) −x, y, −z + 1/2.

The **1** compound crystallizes in a monoclinic system in the *C*2/c space group. The molecule lies on a two-fold axis. The core of the compound resembles a distorted cube and consists of four lithium atoms bonded to four oxygen atoms derived from triphenylsiloxide ligands. Additionally, two metal atoms coordinate two THF molecules (one of each) that are not part of the core. The molecular structure of **1** is shown in Figure 1, and the selected bond lengths and angles are given in Table 1.

Two crystallographically independent lithium atoms in **1** have different coordination numbers, which probably influences the shape of the molecule’s core. The Li1 atom is additionally bound to the oxygen atom of the THF molecule, which affects the extension of Li-O bonds with the Ph_3_SiO^−^ anion in relation to the Li2 atom. The lengths of the metal-oxygen bonds with the oxygen atoms of the triphenylsiloxide ligand for Li1 are about 2.00 Å, while for Li2, ca. 1.9 Å. The lengths of M-O bonds with oxygen atoms O1 and O2 are close to the length of the bonds in the crystal [Li(DME)(OSiPh_3_)]_2_ [7], which are in the range of 1.881(10)–1.910(9) Å. The length of all Si-O and Si-C bonds is also similar to those in the previously mentioned compound [7]. For literature molecule, Si-O and Si-C bonds are in the following ranges: 1.6032(9)–1.6056(9) Å and 1.8797(14)–1.8944(13) Å, while for the described **1** structure, their values stay between 1.6055(9)–1.6056(9) Å and 1.8797(14)–1.8945(14) Å, respectively. The structure of [Li(DME)(Ph_3_SiO)]_2_ resembles the asymmetric part occurring in **1**. However, in the reported case, all-metal atoms are additionally bound to oxygen atoms from THF that are not part of the molecule’s core.

**Figure 2:**
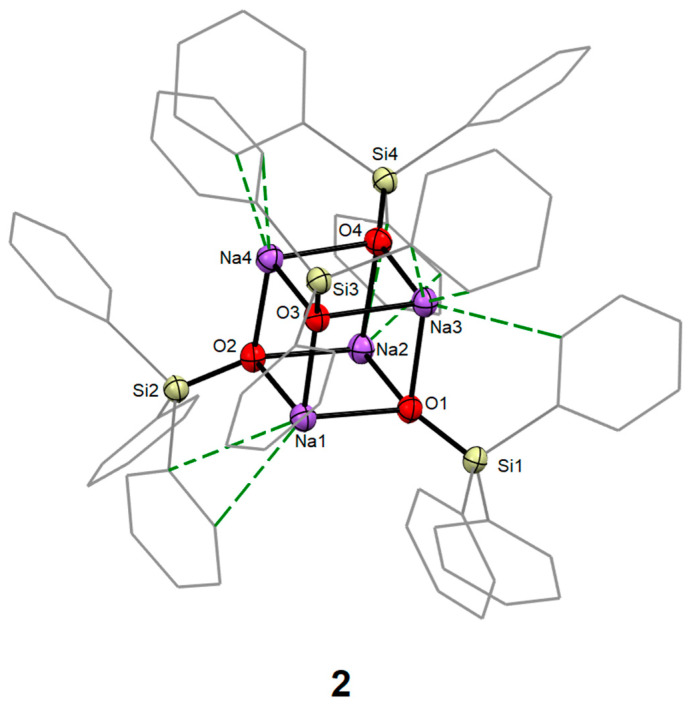
Molecular structure of **2** (50% probability ellipsoids). Hydrogen atoms are omitted for clarity. Dashed green lines indicate intramolecular Na-π interactions.

In turn, compound **2**, shown in Figure 2, crystallizes in a triclinic system in the *P*-1 space group.

The structure of **2** consists of four sodium atoms connected to four oxygen atoms, which form the core of the molecule. All oxygen atoms in **2** belong to triphenylsiloxide ligands. Similarly to **1**, this metal-oxygen core has a cube geometry with alternating sodium and oxygen atoms at the corners. Selected values of bond lengths and angles of **2** are presented in Table 1.

The lengths of the Na-O bonds in **2** are in the range of 2.208(2)–2.346(2) Å, which correspond to the values in the [Na_4_(OSiPh_3_)_4_(H_2_O)_3_] crystal [10]. The same is observed for Si-O and Si-C bonds, which range from 1.586(2)–1.591(2) and 1.880(4)–1.907(3) Å, whereas, in **2**, these bond lengths are equal to 1.587(2)–1.589(2) Å and 1.880(3)–1.908(3) Å, respectively. In turn, the angles between the atoms making up the core have values close to 90°, which indicates an almost perfect cubic geometry of the sodium-oxygen core.

Besides the Na—O bonds in **2**, supramolecular π-interactions between sodium cations and phenyl rings of Ph_3_SiO^−^ ligands are also observed. Metal atoms are connected to two or three carbon atoms from phenyl rings. Na1 and Na2 are bonded to two carbon atoms in one phenyl ring, while Na3 and Na4 are bonded to three carbon atoms from different phenyl rings. The lengths of the Na-C bonds are in the range 2.796(4)–3.088(4) Å. The wide range of this value is due to the different arrangement of the phenyl rings in relation to Na atoms in space. π-interactions between metal atoms and phenyl rings occur also in other compounds containing triorganosilanol ligands such as [NaOSiMePh_2_]_6_, [NaOSiMePh_2_]_4_(THF)_4_ [25], and [NaOSi(*t*Bu)_2_Ph]_4_ [26]. Na-C bonds in these structures have lengths in ranges of 2.809(7)–3.024(7) Å, 2.731 (2)–2.922(2) Å, and 2.925(9)–2.990(7) Å, respectively. These values are similar to each other and those found in **2**.

**Scheme 2:**
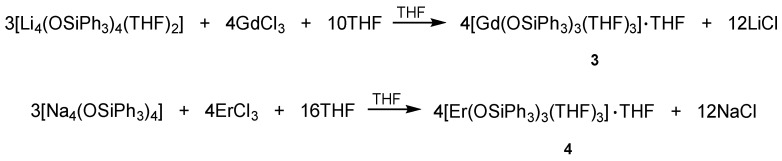
Synthesis reaction scheme of **3** and **4**.

The resulting **1** and **2** triphenysiloxides were used in the next step toward the synthesis of gadolinium [Gd(OSiPh_3_)_3_(THF)_3_]∙THF (**3**) and erbium [Er(OSiPh_3_)_3_(THF)_3_]∙THF (**4**) coordination entities as shown in Scheme 2. Similar compounds with lanthanide metals were obtained by reaction with metal nitrates and “NaOSiPh_3_(THF)” [15]. X-ray investigations confirmed their crystals structures. It should be noted here that the structure of [Er(OSiPh_3_)_3_(THF)_3_]∙THF (**4**) has already been described [27], but it was determined at 203 K than in the reported approach in this paper.

**Figure 3:**
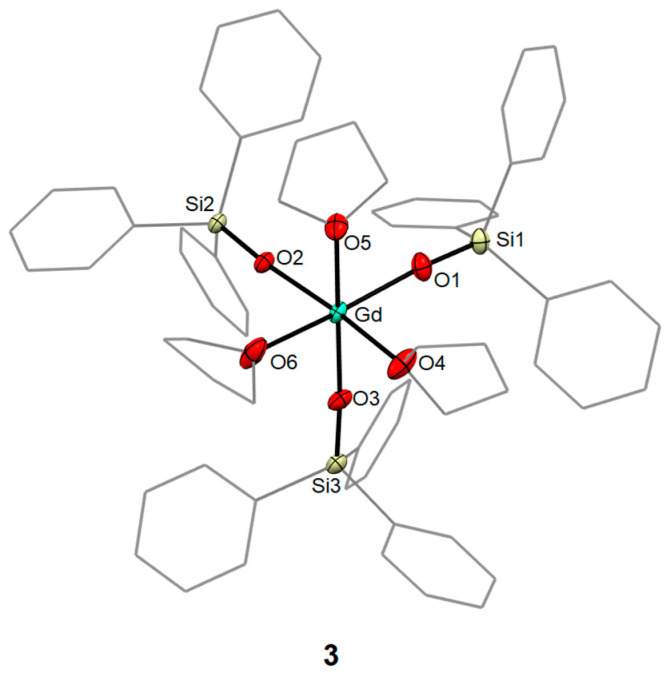
Molecular structure of **3** (50% probability ellipsoids). Hydrogen atoms are omitted for clarity.

In the case of lanthanide(III) triphenylsiloxides, the X-ray analysis of single crystals revealed that both crystal are isomorophous and crystallize in a monoclincic system in the *P*2_1_ space group. Molecular structure of **3** is presented in Figure 3.

Both structures consist of a six-coordinated metal cation (Gd^3+^ and Er^3+^ for 3 and 4, respectively), bound to six oxygen atoms. The oxygen atoms belong to three triphenylsiloxide ligands and three THF molecules.

Molecules show the geometry of the distorted octahedron manifesting the *fac* isomerism; it is related to the arrangement of ligands, where three Ph_3_SiO^−^ anions occupy adjacent positions at the corners of the octahedron, similarly to THF molecules. In the crystal structure of **3** and **4**, apart from the coordination entities, there is also a solvated THF molecule.

Compounds **3** and **4** constitute isomorphic structures of the general formula [M(OSiPh_3_)_3_(THF)_3_]∙THF (M = Gd^3+^, Er^3+^) not only concerning each other but also with the other lanthanide complexes already described in the literature, such as yttrium(III), cerium(III), praseodymium(III) [15], lanthanum(III) [16], samarium(III) [28], and dysprosium(III) [29]. All mentioned above compounds have the same octahedral geometry as gadolinium and erbium molecules described in this paper. Selected bond lengths for **3** and **4** are presented in Table 2.

The lengths of metal-oxygen bonds with oxygen atoms of Ph_3_SiO^−^ anion in **3** and **4** are comparable, and their values equal approx. 2.1 Å. The same relation is observed with the M-O bonds between metal center and THF molecules; the lengths of these bonds are approx. 2.4 Å. These values also correspond to those M-O distances of the lanthanide compounds given above, which also possess a distorted octahedron geometry [30]. The values of the O-M-O angles are also evidence of the distorted structure occurring in **3** and **4**. In a perfect octahedron, the angles between the vertices and the center of the polyhedron are 90 and 180°. In molecules **3** and **4**, values of angles between Ph_3_SiO^−^ ligands and metal center are slightly larger than 90°, and angles between THF ligands and metal center are slightly smaller than 90°.

### 3.2. IR and Raman Spectroscopy of **3** and **4**

Looking at the articles published so far on compounds with the general formula [M(OSiPh_3_)_3_(THF)_3_]∙THF (M = lanthanide ion), it is surprising that the research was focused only on the crystal structures of these compounds, without delving into, inter alia, their spectroscopic properties. At least from this point of view, supplementing this knowledge is necessary due to the fact that no one has paid attention to these issues. Resulting **3** and **4** species were additionally examined by Raman and IR spectroscopies. Since both compounds are sensitive and react with the solid dielectric medium, the ATR technique was applied instead of the standard measurement in a KBr pellet.

Crystals of **3** and **4** adopt the *P*2_1_ monoclinic symmetry with two formula units in a primitive cell; therefore, the total number of vibrational modes, predicted by group theory, equals 948. Since all atoms are located in the general 2a position (C_1_ site), the total irreducible representation is 474A + 474B. All vibrational Brillouin zone center modes can be further subdivided into 315A + 315B internal modes, 9A + 9B translations, and 9A + 9B librations of the Ph_3_SiO^−^ ions, as well as 156A + 156B internal modes, 12A + 12B translations, and 12A + 12B librations of THF molecules. The lanthanide ions give rise to 3A + 3B translational lattice modes. Three of them (A + 2B) belong to the acoustic branch and are not detectable by vibrational spectroscopy. The remaining 473A + 472B are optical modes; they are simultaneously IR- and Raman-active. The comparison of simplified selection rules for the **3** and **4** crystals and ligands (both Ph_3_SiO^−^ and THF) is presented in Table S3 (Supplementary Materials).

**Figure 4:**
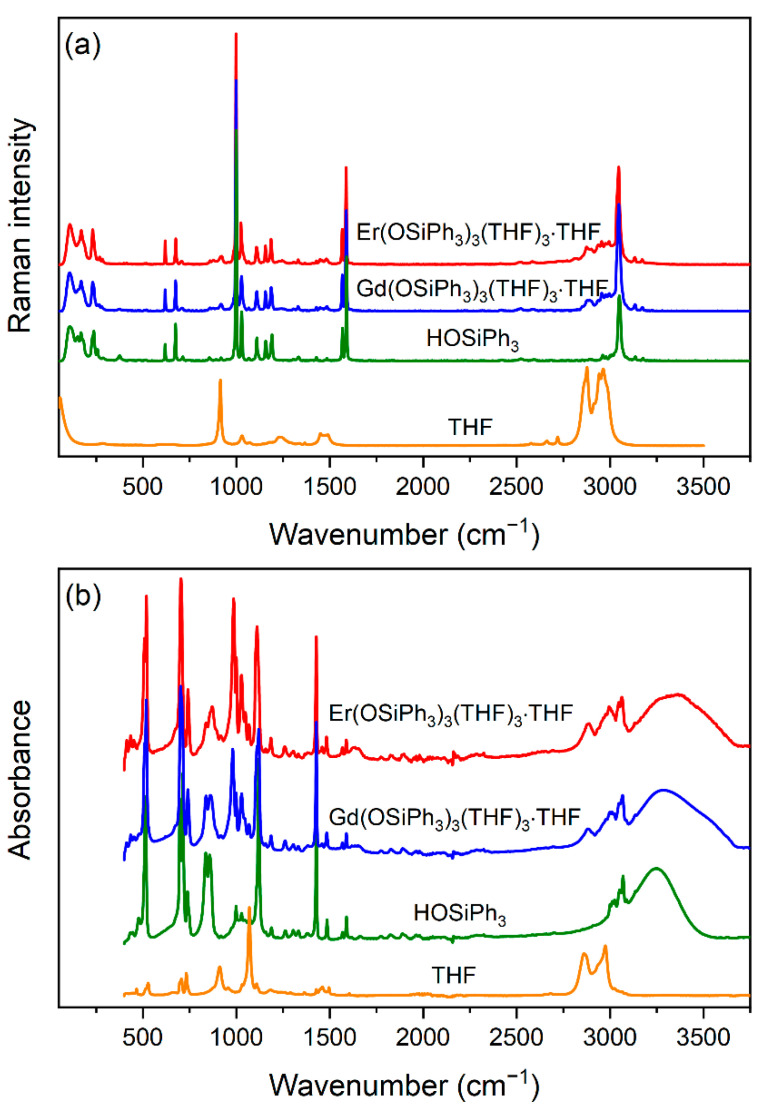
Raman (**a**) and IR (**b**) spectra of **3**, **4**, Ph_3_SiOH, and THF.

Figure 4 presents the measured IR and Raman spectra compared to the spectra of pure ligands, liquid THF, and polycrystalline Ph_3_SiOH. Table S4 lists the observed Raman and IR wavenumbers together with the proposed assignments. It should be noted that the number of observed vibrational bands for the complexes is significantly lower than theoretically predicted. Such a low magnitude of Davydov (factor group) splitting suggests a weak intermolecular interaction in the studied materials. Nevertheless, the vibrational spectra of both compounds are very similar, indicating the same crystal architecture.

The internal vibrations of THF, i.e., ν_s_(CH_2_) and ν_as_(CH_2_), the symmetric and antisymmetric CH_2_ stretching, respectively; ν(CC), ν(CO) the ring stretching; δ(CC), δ(CO) the ring bending; β(CH) and γ(CH), the in-the-plane and out-of-plane bending, respectively, were assigned based on the previous experimental data and reported DFT calculations [31,32]. All characteristic THF bands can be easily located in the spectra of **3** and **4**. For instance, the ν_s_(CH_2_) and ν_as_(CH_2_) bands are observed for the pure THF as two broad and intense contours in the 2860–2876 cm^−1^ and 2916–2984 cm^−1^ ranges, respectively. They correspond to IR and Raman bands observed for **3** and **4** in the 2852–2889 cm^−1^ and 2910–2978 cm^−1^ ranges. The presence of IR (Raman) bands corresponding to the γ(CH) modes, observed for **4** at 1458 cm^−1^ (1459–1460 cm^−1^) and for **3** between 1447 and 1449 cm^−1^ (at 1448 cm^−1^), is further proof of THF complexation.

The low shifts of bands when the THF molecules are coordinated or accommodated in the crystal voids, in comparison to the liquid phase, suggest that the conformation of this ligand is weakly affected in crystals.

IR and Raman spectra of the second ligand, triphenylsilanol Ph_3_SiOH, are very similar to the spectra of the studied crystals **3** and **4**. Therefore, there is no doubt that the Ph_3_SiO^−^ ions are present in the studied complexes and that their geometry is comparable to that observed in the pure crystal of triphenylsilanol. The change of intensity and vanishing of some lattice modes (below 200 cm^−1^), observed for **3** and **4**, evidence that the triphenylsiloxide anions are coordinated to the lanthanide cations. The IR spectrum of Ph_3_SiOH exhibits a symmetric, strong, and broad absorption band with a maximum at 3247 cm^−1^ originating from the stretching vibrations of hydroxyl groups, ν(OH). This band is also observed in the IR spectra of **3** and **4**, with a second broad component with a maximum of about 3380 cm^−1^. Since our X-ray diffraction data did not show the presence of water molecules in the crystal, these broad bands originate more likely from the residuals of the ligands on the surface of the crystals and/or adsorbed water. The occurrence of weak and broad bands observed near 1640 cm^−1^ for both complexes supports the presence of adsorbed water since this is a typical range of the δ(OH) bending modes.

The assignment of characteristic bands of triphenylsilanol, mainly corresponding to the phenyl rings, is based on the previously reported experimental IR and Raman data [33]. The internal vibrations involving Si^4+^ ions, the ν(OSiC) stretching, the δ(SiO) and δ(SiOC) bending modes, were found in the characteristic narrow ranges, i.e., in the 1047–1157 cm^−1^, 741–922 cm^−1^, and 413–481 cm^−1^ ranges, respectively. They were previously reported for Ph_3_SiOH and other alkyl and aryl silanols [33,34]. The small shifts of the listed bands after complexation suggest that the geometry of the SiO_4_ tetrahedra is very similar in the ligand and **3** and **4** crystals.

T’(Ln^3+^) translations of lanthanide ions contribute to the lattice modes between 250 and 300 cm^−1^. It should be noted that the substitution effect of the metal ion to the position of Raman bands is weak since the mass and ionic radii of Er^3+^ and Gd^3+^ in the octahedral coordination are comparable, 103.0 and 107.8 pm, respectively. Furthermore, the Gd-O distances in the large and distorted GdO_6_ octahedra are relatively long, namely 2.1457–2.4965 Å.

### 3.3. Absorption Spectra of **3** and **4**

**Figure 5:**
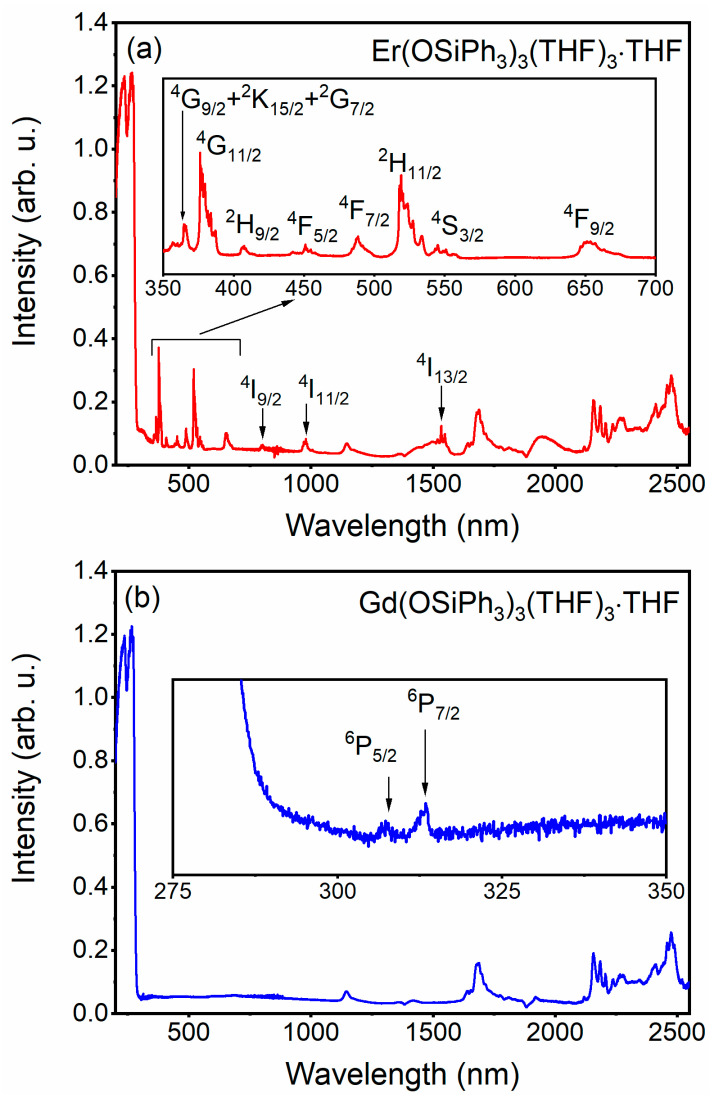
The absorption spectra of **4** (**a**) and **3** (**b**).

The absorption spectra of **3** and **4** measured at RT in the range of 200–2500 nm are presented in Figure 5. A broad and intense band in the ultraviolet region, as well as a few less intense transitions in the near-IR range, are observed and attributed to the Ph_3_SiOH ligand absorption (see Figure S1). The peaks located in the near-IR region (2117–2233 nm and 2412–2520 nm) correspond mainly to combinational bands and some overtones of the ν(CH_2_), ν(CH) stretching and the β(CH_2_), γ(CH_2_), and δ(CH) bending modes. The first and second overtones of the CH_2_/CH stretching can be ascribed to the bands observed in the 1638–1711 nm range and at 1143 nm, respectively. The spectra of **3** and **4** also exhibit two broader bands located at 1506 and 1948 nm originating from the ν + δ(OH) combination of stretching and bending modes, and the second stretching overtone, 2ν(OH), respectively. This observation agrees with ATR spectra which confirmed the presence of adsorbed water in **3** and **4**. It should be noted that the intensity of water-related absorption bands is higher for the erbium analog, as was observed in the ATR spectra.

The f-f bands characteristic for Er^3+^ ions are located at 365 nm (^4^G_9/2_ + ^2^K_15/2_ + ^2^G_7/2_), 375 nm (^4^G_11/2_), 407 nm (^2^H_9/2_), 451 nm (^4^F_5/2_), 488 nm (^4^F_7/2_), 518 nm (^2^H_11/2_), 545 nm (^4^S_3/2_), 652 nm (^4^F_9/2_), 800 nm (^4^I_9/2_), 980 nm (^4^I_11/2_), and 1532 nm (^4^I_13/2_) [35,36], while bands typical for Gd^3+^ ions are observed at 307 nm (^6^P_5/2_) and 313 nm (^6^P_7/2_) [37], see Figure 5a,b, respectively. The absorption spectra were processed to calculate the energy bandgap (E_g_) of the studied compounds according to the Kubelka–Munk theory using the following equation [38]:

(1) $$F\left(R\right)=\frac{{\left(1-R\right)}^{2}}{2R}$$

where *R* is the reflectance. The E_g_ of investigated materials were determined to be 4.45 eV regardless of the lanthanide ion present in the crystal structure (Figure S2).

### 3.4. Magnetic Properties of **3** and **4**

In lanthanides, 4f orbitals are efficiently shielded, and the influence of the neighboring groups on the magnetic properties is less evident than in 3d paramagnetic compounds. The difficulty in studying the magnetic properties of compounds containing Ln^3+^ ions arises from their orbital momentum. For a 4f^n^ configuration, it can result in up to 2*J* + 1 Stark levels if *n* is even and *J* + 1/2 levels if n is odd. Suppose the Ln^3+^ ion is in exchange with another paramagnetic species. In that case, two cumulative phenomena occur, one being related to interactions between the two magnetic centers. At the same time, the second one is intrinsic to the lanthanide ion and is due to the thermal population of the Stark sub-levels. For the f-elements, the ligand field perturbation splits the spectroscopic level ^2S+1^L_J_ of the metal ion. When the temperature decreases, the effective magnetic moment of the Ln^3+^ ion changes by thermal depopulation of the Stark sub-levels, and the magnetic behavior of the Ln^3+^ ion deviates concerning the Curie law. This phenomenon is modulated by the ligand field and the symmetry of the compound. Thus, in complexes containing lanthanide ions with configurations other than f^7^ (or f^0^ or f^14^), the combined effects of first-order angular momentum and the ligand field lead to the anisotropy of the magnetic susceptibility. Thus the analysis of any exchange interactions becomes a formidable task. Only the Gd^3+^ ion does not possess a first-order orbital moment, and the anisotropic effect does not have to be considered [39,40,41,42].

**Figure 6:**
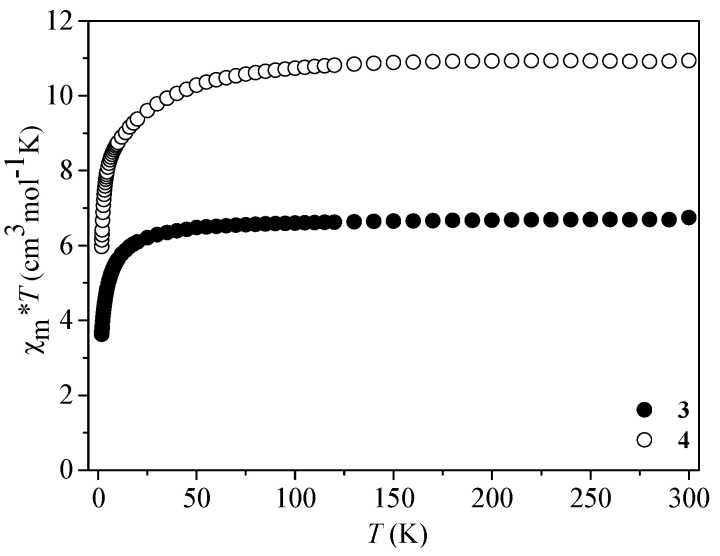
Temperature dependence of experimental *χ_m_T* for **3** (●) (*χ_m_* per Gd^3+^ ion) and **4** (○) (*χ_m_* per Er^3+^ ion).

Under an applied field of 0.5 T, direct-current (dc) magnetic susceptibility measurements of compounds **3** and **4** were performed on polycrystalline samples during the temperature range of 1.8–300 K. Plots of *χ_m_T* product versus *T* (*χ_m_* is the molar magnetic susceptibility per Ln^III^ ion) for **3** and **4** are given in Figure 6. The experimentally determined values of *χ_m_T* of investigated compounds at RT were compared with the theoretical values of *χ_m_T* calculated by the equation *χ_m_T* = ((*Nβ*^2^/3*k*)[*g*^2^*_Ln_J_Ln_*(*J_Ln_* + 1)]), where *N* is Avogadro constant, *β* is the Bohr magneton, *k* is Boltzman’s constant, and *g_Ln_* is the *g* factor of the ground *J* terms of Ln^3+^ expressed as [43]:

(2) $$g_{Ln}^{}=\frac{3}{2}+\frac{S(S+1)-L(L+1)}{2J(J+1)}$$

The room temperature *χ_m_T* value of 6.75 cm^3^ K mol^−1^ for **3** is slightly lower than the expected *χ_m_T* value (7.88 cm^3^ K mol^−1^) for a magnetically dilute Gd^3+^ ion (^8^S_7/2_, *J* = 7/2, *L* = 0, *S* = 7/2, *g* = 2.0). The *χ_m_T* product remains almost constant as the temperature is decreased from 300 to 30 K, before a sharp decrease below this temperature to 3.63 cm^3^ mol^−1^ K at 1.8 K.

The decline of *χ_m_T* at lower temperatures may be attributed to the zero-field splitting (ZFS) and/or Zeeman depopulation effects or the weak intermolecular antiferromagnetic interaction between Gd^3+^ ions of neighboring molecules. The contribution of the ^8^S_7/2_ ground state of Gd^3+^ ion to the observed single-ion magnetic anisotropy in **3** is likely to be negligible because of the absence of first-order orbital angular momentum. The presence of the anisotropy may arise from admixture of the ground state to the low-lying excited states, where unquenched orbital angular momentum can significantly contribute to the single-ion anisotropy of the Gd^3+^ ion, or the crystal field and spin-spin/dipolar interactions. Several recent reports show that the dipolar contribution is the main factor in the observed magnetic anisotropy of Gd^3+^ systems [43,44]. At 300 K, the *χ_m_T* product of **4** equals 11.09 cm^3^ mol^−1^ K which roughly corresponds to the value expected for the isolated Er^3+^ ion (^4^I_15/2_, *J* = 15/2, *S* = 3/2, *L* = 6, *g* = 6/5) (11.48 cm^3^ Kmol^−1^). Upon cooling, the *χ_m_T* is practically constant down to 100 K, after which it begins a steeper reduction of the *χ_m_T* to 6.06 cm^3^mol^−1^ K at 1.8 K. Such behavior of **4** generally can be attributed to the combined effect of thermal depopulation of the Stark levels modulated by the crystal field of Er^3+^ ions and/or a weak intermolecular antiferromagnetic interaction [39,45]. Additionally, no alternating current (ac) magnetic susceptibility signals were observed in the absence and the presence of applied static fields in **3** and **4**.

The thermal evolution of χ_m_^−1^ obeys the Curie–Weiss law in the whole temperature range. A fit of χ_m_^−1^ to the Curie–Weiss law gives Curie constants (C) of 6.74 cm^3^Kmol^−1^ (**3**) and 11.03 cm^3^Kmol^−1^ (**4**) and Weiss temperatures (θ) of −1.93 K (**3**) and −2.39 K (**4**). The negative and low parameters θ of **3** and **4** may indicate weak antiferromagnetic interactions between the neighboring lanthanide(III) ions in the crystal lattice.

**Figure 7:**
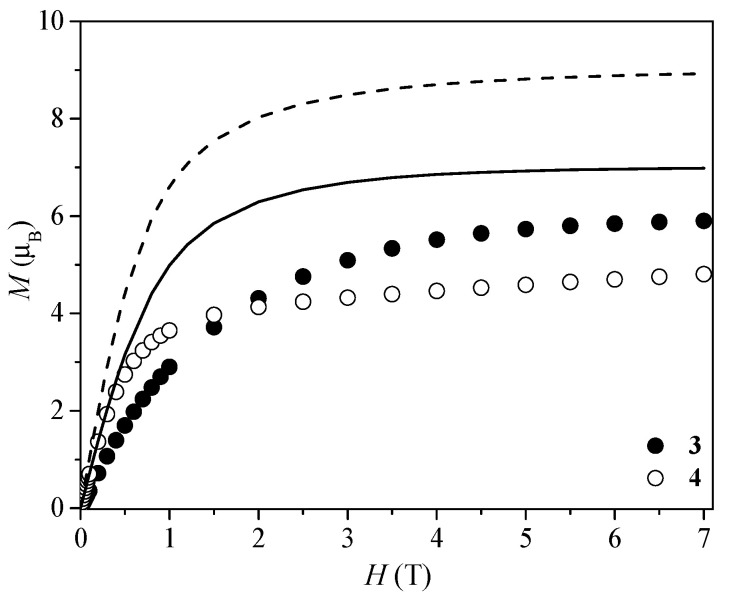
Field dependence of the magnetization for **3** (●) (*M* per Gd^3+^ ion) and **4** (○) (*M* per Er^3+^ ion) at 2 K. The solid line is the Brillouin function curve for the system of one independent *J* = ^7^/_2_; the dashed line is the Brillouin function curve for the system of one independent *J* = ^15^/_2_.

The nature of the ground state of **3** and **4** was verified by isothermal magnetization studies at 2 K (Figure 7). The M versus H plot for **3** shows a slow increase with applied field to reach magnetization value of ~6 μ_B_ at 7 T. However, this saturation value for the highest fields does not precisely match the *J* = 7/2 ground state.

This further may indicate that the sharp decrease of *χ_m_T* at low temperatures may occur due to intermolecular and/or Zeeman effects. Considering the quite long intermolecular distance between the closest Gd···Gd distances (above 11 Å), it seems unlikely that the intermolecular interactions will have a significant influence on the magnetic behavior. Conversely, compound **4** does not reach saturation with a magnetization value of ~5 μ_B_ at 7 T. Such behavior may reflect both the intermolecular interactions and the magnetic anisotropy and significant crystal field effects from the Er^3+^. Unfortunately, the quantitative description of the magnetic properties of complexes containing lanthanide(III) ions is not an easy task because of the ligand-field effect and spin-orbit coupling of the Ln^3+^ ion [41,42].

## 4. Conclusions

Triphenylsiloxides of alkali metals (lithium and sodium, **1** and **2**, respectively) and trivalent lanthanides (gadolinium and erbium, **3** and **4**, respectively) have been synthesized and fully characterized by X-ray crystallography. IR and Raman spectroscopy confirmed the formation of **3** and **4** by the presence of characteristic bands corresponding to internal and lattice modes. The assignment of the observed vibrational bands was proposed. It was shown that both **3** and **4** compounds adopt the same crystal architecture and that the conformation of ligands is comparable to observed in triphenylsilanol Ph_3_SiOH crystal and the liquid phase of THF. Furthermore, the geometry of the SiO_4_ tetrahedron is weakly affected after complexation. Vibrational studies exhibited low Davydov splitting and weak intermolecular interactions in both studied compounds. The absorption spectra showed characteristic bands corresponding to the f-f transitions of Er^3+^ and Gd^3+^ ions. The calculated band gap energy for both compounds equals 4.45 eV and has a slightly higher value than that of the triphenylsiloxide ligand (4.34 eV). Both spectroscopic experiments revealed that the studied complexes adsorb water that is expected to quench the emission properties. The magnetic susceptibility measurements and a relationship between the magnetization and magnetic field strength in **3** revealed a nearly isolated mononuclear unit. Magnetic studies of **4** are dominated by the crystal field effect on the Er^3+^ ion.

## Figures and Tables

**Table 1:** Selected bond lengths [Å] and angles [deg] for **1** and **2**.

**1.**	Li1···O1	2.005(2)	Si1···C1A	1.8798(14)
	Li1···O2	2.003(2)	Si1···C1B	1.8945(14)
	Li1···O2^i^	1.996(2)	Si1···C1C	1.8913(14)
	Li1···O3	1.942(2)	Si2···O2	1.6056(9)
	Li2···O1	1.900(2)	Si2···C1D	1.8944(13)
	Li2···O1^i^	1.928(2)	Si2···C1E	1.8797(14)
	Li2···O2	1.890(2)	Si2···C1F	1.8912(13)
	Si1···O1	1.6055(9)	O3···Li1···O2	115.08(11)
	O2···Li1···O1	95.59(10)	O3···Li1···O2^i^	125.10(12)
	O2···Li1···O1^i^	94.59(10)	O2···Li2···O1	103.12(11)
	O2···Li1···O2^i^	96.68(10)	O2···Li2···O1^i^	100.73(11)
	O3···Li1···O1	123.17(12)	O1···Li2···O1^i^	99.60(11)
2	Na1···O1	2.239(3)	Si1···C1B	1.908(3)
	Na1···O2	2.346(3)	Si1···C1C	1.895(3)
	Na1···O3	2.235(3)	Si2···O2	1.589(2)
	Na2···O1	2.231(3)	Si2···C1D	1.895(4)
	Na2···O2	2.209(3)	Si2···C1E	1.881(3)
	Na2···O4	2.331(3)	Si2···C1F	1.904(3)
	Na3···O1	2.307(3)	Si3···O3	1.589(2)
	Na3···O3	2.311(3)	Si3···C1G	1.897(4)
	Na3···O4	2.198(3)	Si3···C1H	1.907(3)
	Na4···O2	2.248(3)	Si3···C1I	1.886(3)
	Na4···O3	2.262(3)	Si4···O4	1.587(2)
	Na4···O4	2.281(3)	Si4···C1J	1.894(3)
	Si1···O1	1.588(3)	Si4···C1K	1.896(4)
	Si1···C1A	1.895(3)	Si4···C1L	1.882(3)
	O1···Na1···O2	90.33(9)	O1···Na3···O3	89.24(9)
	O1···Na1···O3	92.93(9)	O1···Na3···O4	93.45(9)
	O2···Na1···O3	91.99(9)	O3···Na3···O4	92.58(9)
	O1···Na2···O2	94.19(9)	O2···Na4···O3	93.87(9)
	O1···Na2···O4	91.94(9)	O2···Na4···O4	91.59(9)
	O2···Na2···O4	91.25(9)	O3···Na4···O4	91.71(9)

Symmetry code: (i) −x, y, −z + 1/2.

**Table 2:** Selected bond lengths [Å] and angles [deg] for **3** and **4**.

3		4	
Gd···O1	2.170(3)	Er···O1	2.119(8)
Gd···O2	2.157(3)	Er···O2	2.118(9)
Gd···O3	2.146(3)	Er···O3	2.108(8)
Gd···O4	2.461(3)	Er···O4	2.428(9)
Gd···O5	2.497(3)	Er···O5	2.407(9)
Gd···O6	2.478(3)	Er···O6	2.375(9)
O1···Gd···O4	90.24(12)	O1···Er···O4	88.9(3)
O1···Gd···O5	86.47(12)	O1···Er···O5	86.8(4)
O1···Gd···O6	164.51(13)	O1···Er···O6	164.5(4)
O2···Gd···O1	101.37(12)	O2···Er···O1	102.2(4)
O2···Gd···O4	163.71(10)	O2···Er···O4	85.8(3)
O2···Gd···O5	88.23(11)	O2···Er···O5	162.3(4)
O2···Gd···O6	86.42(12)	O2···Er···O6	88.6(4)
O3···Gd···O1	100.83(12)	O3···Er···O1	100.6(3)
O3···Gd···O2	101.42(11)	O3···Er···O2	101.3(4)
O3···Gd···O4	87.37(11)	O3···Er···O4	166.6(3)
O3···Gd···O5	166.36(11)	O3···Er···O5	91.9(4)
O3···Gd···O6	90.56(13)	O3···Er···O6	88.0(3)
O4···Gd···O5	81.05(11)	O5···Er···O4	79.1(4)
O4···Gd···O6	79.74(12)	O6···Er···O4	80.7(3)
O6···Gd···O5	80.35(13)	O6···Er···O5	80.0(4)

Symmetry codes: −x, y + 1/2, −z.

## Data Availability

Not applicable.

## Supplementary Materials

The following are available online. Table S1: Crystallographic data for **1** and **2**. Table S2: Crystallographic data for **3** and **4**. Table S3: The selection rules for the Ln(OSiPH_3_)_3_(THF)_3_·THF (Ln = Er^3+^, Gd^3+^) crystals compared to the Ph_3_SiOH crystal and THF. Table S4: Tentative assignments of the observed vibrational modes for the Ln(OSiPH_3_)_3_(THF)_3_·THF (Ln = Er^3+^, Gd^3+^) crystals and the ligands (HOSiPh_3_ and THF). Figure S1: The absorption spectrum of the Ph_3_SiOH ligand and the result of the energy band gap (E_g_) calculation using the Kubelka-Munk theory. Figure S2: The results of the energy band gap (E_g_) calculation using the Kubelka–Munk theory for the Er(OSiPh_3_)_3_(THF)_3_·THF (a) and Gd(OSiPh_3_)_3_(THF)_3_·THF (b) crystals.

## Sample Availability

Samples of the compounds are available from the authors.
