# Supplementary material for: Synthesis, Crystal Structures, and Spectroscopic Properties of Novel Gadolinium and Erbium Triphenylsiloxide Coordination Entities
Source: Molecules. 2021 Dec 27;27(1):147. doi: 10.3390/molecules27010147 (PMC8746333; doi:10.3390/molecules27010147)
Supplement: Supplementary file 1 [file molecules-27-00147-s001.zip › molecules-1520313-supplementary.pdf]

Supplementary Materials

Synthesis, crystal structures and spectroscopic properties of novel gadolinium and erbium triphenylsiloxide coordination entities

Patrycja Wytrych[1], Józef Utko[1], Julia Kłak[1], Maciej Ptak[2], Mariusz Stefanski[2], Tadeusz Lis[1], Jolanta Ejfler[1] and Łukasz John*[,1]

[1] Faculty of Chemistry, University of Wrocław, 14 F. Joliot-Curie, 50-383 Wrocław, Poland
[2] Institute of Low Temperature and Structure Research, Polish Academy of Sciences, 2 Okólna, 50-422 Wrocław, Poland

*Cooresponding author: Dr. Łukasz John, D.Sc., e-mail: lukasz.john@chem.uni.wroc.pl.

Table S1. Crystallographic data for **1** and **2**.

	**1**	**2**
Chemical formula	$C_{80}H_{76}Li_4O_6Si_4$	$C_{72}H_{60}Na_4O_4Si_4$
M_r	1273.52	1193.52
Crystal system, space group	Monoclinic, *C*2/*c*	Triclinic, *P*-1
Temperature (K)	100	100
a, *b*, *c* (Å)	22.030 (4), 13.942 (2), 23.354 (4)	14.486 (4), 14.859 (4), 15.223 (4)
α, β, γ (°)	90, 108.53 (2), 90	79.45 (2), 81.28 (3), 82.72 (3)
V (Å^3)	6801 (2)	3167.5 (15)
Z	4	2
Radiation type	Cu *K*α	Cu *K*α
μ (mm^{-1})	1.23	1.53
Crystal size (mm)	0.22 × 0.14 × 0.10	0.45 × 0.37 × 0.11
Data collection		
Diffractometer	Xcalibur, Ruby, Gemini ultra	Xcalibur, Onyx
Absorption correction	Analytical	Analytical
T_{min}, T_{max}	0.837, 0.901	0.653, 0.885
No. of measured, independent and	14419, 5902, 5539	25717, 12578, 9882
R_{int}	0.016	0.074
$(\sin \theta/\lambda)_{max}$ (Å^{-1})	0.599	0.627
Refinement		
$R[F^2 > 2\sigma(F^2)]$, $wR(F^2)$, S	0.030, 0.081, 1.02	0.083, 0.238, 1.05
No. of reflections	5902	12578
No. of parameters	424	757
H-atom treatment	H-atom parameters constrained	H-atom parameters constrained
$\Delta\rho_{max}$, $\Delta\rho_{min}$ (e Å^{-3})	0.37, −0.30	0.96, −0.64

Computer programs: *CrysAlis PRO* 1.171.39.46 (Rigaku OD, 2018), *SHELXS97* (Sheldrick, 1990), *SHELXL2013* (Sheldrick, 2013).

Table S2. Crystallographic data for **3** and **4**.

	**3**	**4**
Chemical formula	$C_{66}H_{69}GdO_6Si_3{\cdot}C_4H_8O$	$C_{66}H_{69}ErO_6Si_3{\cdot}C_4H_8O$
M_r	1271.83	1281.84
Crystal system,	Monoclinic, $P2_1$	Monoclinic, $P2_1$
Temperature (K)	100	100
a, *b*, *c* (Å)	14.335 (4), 16.482 (4), 14.569 (4)	14.489 (5), 16.422 (4), 14.378 (5)
β (°)	113.82 (2)	113.83 (4)
V (Å^3)	3149.0 (15)	3129.4 (19)
Z	2	2
Radiation type	Mo $K\alpha$	Mo $K\alpha$
μ (mm^{-1})	1.16	1.45
Crystal size (mm)	0.37 × 0.31 × 0.09	0.29× 0.09 × 0.06
Data collection		
Diffractometer	Ruby	Kuma KM4 kappa-geometry diffractometer with a CCD detector
Absorption	Analytical	Analytical
T_{min}, T_{max}	0.730, 0.908	0.747, 0.928
No. of measured,	26868, 16403, 14946	17872, 8659, 7138
R_{int}	0.029	0.089
$(\sin \theta/\lambda)_{max}$ (Å^{-1})	0.718	0.606
Refinement		
$R[F^2 > 2\sigma(F^2)]$,	0.034, 0.073, 1.03	0.063, 0.148, 1.15
No. of reflections	16403	8659
No. of parameters	730	719
No. of restraints	1	1
H-atom treatment	H-atom parameters constrained	H-atom parameters constrained
$\Delta\rho_{max}$, $\Delta\rho_{min}$ (e Å^{-3})	1.13, −0.70	1.68, −2.53
Absolute structure	Flack x determined using 6104 quotients [(I+)-(I-)]/[(I+)+(I-)] (Parsons, Flack and Wagner, Acta Cryst. B69 (2013) 249-259).	Flack x determined using 1773 quotients [(I+)-(I-)]/[(I+)+(I-)] (Parsons and Flack (2004), Acta Cryst. A60, s61).
Absolute structure parameter	−0.029 (3)	0.033 (15)

Computer programs: SHELXL2018/1 (Sheldrick, 2018), R.C. Clark & J.S. Reid. (Clark, R. C. & Reid, J. S. (1995). Acta Cryst. A51, 887-897), *CrysAlis PRO*, Agilent Technologies, Version 1.171.36.20 (release 27-06-2012 CrysAlis171 .NET) (compiled Jul 11 2012, 15:38:31), *SHELXS97* (Sheldrick, 1990), *SHELXL2013* (Sheldrick, 2013).

Table S3. The selection rules for the $Ln(OSiPH_3)_3(THF)_3{\cdot}THF$ (Ln= Er^{3+}, Gd^{3+}) crystals compared to the $HOSiPh_3$ crystal and THF

	**Raman-active modes**	**IR-active modes**
$HOSiPh_3$ ($P\overline{1}$)	$864A_g$ (864)	$861A_u$ (861)
THF (C_{2v})*	$10A_1 + 7A_2 + 9B_1 + 7B_2$ (33)**	$10A_1 + 9B_1 + 7B_2$ (26) **
$Ln(OSiPh_3)_3(THF)_3{\cdot}THF$<br>Ln=$Er^{3+}$, Gd^{3+} ($P2_1$)	473A + 472B (945)	473A + 472B (945)

[a] Key: * liquid phase; ** internal modes only

Table S4. Tentative assignments of the observed vibrational modes for the $Ln(OSiPH_3)_3(THF)_3{\cdot}THF$ (Ln=Er^{3+}, Gd^{3+}) crystals and the ligands ($HOSiPh_3$ and THF)[a]

**Raman (cm^{-1})**				**IR-ATR (cm^{-1})**				**Assignment**
**Er^{3+}**	**Gd^{3+}**	**$HOSiPh_3$**	**THF**	**Er^{3+}**	**Gd^{3+}**	**$HOSiPh_3$**	**THF**	
				3377b	3382b			ν(OH)
				3272b	3285b	3247b		ν(OH)
3172vw	3173vw	3176vw						ν(CH)
3132vw	3134vw	3136vw		3132m	3135m	3136m		ν(CH)
				3082m	3086m	3088m		ν(CH)
				3063m	3067m	3069m		ν(CH)
3046s	3046s	3050m		3046m	3048m	3051m		ν(CH)
3037sh	3037sh			3037sh	3037sh			ν(CH)
				3023m	3023m	3025m		ν(CH)
3011vw	3012vw	3012vw		3010m	3011m	3012m		ν(CH)
2994w	2995w	3000v		3002m	2999m	3001m		ν(CH)
				2995m				ν(CH)
2976w	2977w	2978vw	2984sh	2978m	2978m	2980sh	2974s	ov/cb+$\nu_{as}(CH_2)^*$
2954w	2956w	2961vw	2964vs	2964m	2964sh	2962sh		ov/cb+$\nu_{as}(CH_2)^*$
2937w	2937w		2942vs	2936m	2935w		2940sh	$\nu_{as}(CH_2)^*$
			2916m	2910sh	2910sh	2910w		ov/cb+$\nu_{as}(CH_2)^*$
2900w	2899w	2902vw						ov/cb+$\nu_{as}(CH_2)^*$
	2889w	2891vw						ov/cb+$\nu_s(CH_2)^*$
2874w	2879w		2876vs	2885m	2883w		2871sh	$\nu_s(CH_2)^*$
2852vw	2852w	2851vw	2864sh				2860s	ov/cb+$\nu_s(CH_2)^*$
			2719w					ov/cb
			2661w	2695vw	2696vw	2698vw	2681vw	ov/cb
							2654vw	ov/cb
2584vw	2589vw		2578vw					ov/cb
2518vw	2519vw							ov/cb
2415vw	2418vw							ov/cb
				1892vw	1891vw	1889vw		ov/cb
				1829vw	1827vw	1824vw		ov/cb
				1773vw	1774vw	1775vw		ov/cb

				1653b	1654b	1664vw		ν(CC)
				1629b	1627b	1617vw		ν(CC)
1587s	1588s	1589s		1589w	1589m	1589m		ν(CC)
1566m	1567m	1568m		1567w	1567w	1567w		ν(CC)
1482vw	1484vw	1485vw	1490w	1482w	1484w	1485w	1495w	ν(CC)+γ(CH)*
			1478w					γ(CH)*
1459vw	1460vw			1458w	1458w		1460w	γ(CH)*
1448vw	1448vw		1450w	1449w	1447w		1450sh	γ(CH)*
1428vw	1428vw	1428vw		1429s	1428s	1428w	1428w	ν(CC)+γ(CH)*
				1382vw	1383w	1380w		ov/cb+β(CH)*
1361vw			1366vw	1367w	1368sh		1365w	β(CH)*
1331vw	1330vw	1333vw	1335vw	1332vw	1331w	1333w	1333vw	β(CH)+β(CH)*
1307vw	1305vw	1306vw		1305w	1305w	1304w		ov/cb
				1296w	1296sh		1290vw	β(CH)*
				1260w	1261w	1263w	1259vw	β(CH)+β(CH)*
1245vw	1243vw		1242w					β(CH)*
1231vw			1230w					β(CH)*
1186w	1186w	1191w	1183sh	1185w	1186w	1188w	1182w	β(CH)+γ(CH)*+β(CH)*
1151w	1156w	1157w		1157w	1157w	1156w		β(CH)+δ(SiO)
		1118sh		1119sh	1120s	1120vs	1116sh	β(CH)+β(CH)*+γ(CH)*+δ(CO)*
1108w	1109w	1109w		1111s	1112s	1109sh	1108w	ov/cb+β(CH)*+γ(CH)*+δ(CO)*
				1104sh	1104sh			δ(SiO)
1066vw	1067vw		1071vw	1068m	1068w	1070w	1069vs	δ(SiO)+γ(CH)*
				1049m	1045m	1047vw		δ(SiO)
1025m	1029m	1028w	1030w	1027m	1028m	1028vw	1031w	β(CH)+ν(CO)*+ν(CC)*+β(CH)*
	1025sh							β(CH)
998vs	998vs	998vs		1000s	999m	999w		β(CC)
986sh	986w	986w		985vs	981s	974w	957w	γ(CH)+ν(CO)*+γ(CH)*
922w	918w	918vw	915s	917m	917m	917m	911m	ν(OSiC)+γ(CH)+ν(CC)*+β(CH)*
915sh								ν(OSiC)
				895w	895w	896vw		ν(OSiC)
878w	876w			872m	866sh	862sh		ν(OSiC)
860w	859w	856vw		861m	860m	857m		ν(OSiC)
				839m	838m	836m		ν(OSiC)
				741m	741m	748sh		ν(OSiC)
						739m	732m	γ(CH)+ν(CC)*+δ(CO)*+γ(CH)*
	712vw	713vw				713vs		ν(CC)
707vw	707vw				707sh		706m	ν(CC)*+δ(CO)*+γ(CH)*
675w	675w	675m		703vs	702vs	698vs	696m	γ(CH)+δ(CO)*+γ(CH)*
			656vw				661vw	δ(CO)*+γ(CH)*
620w	619w	619w	600vw					δ(CO)*+γ(CH)*
							527w	δ(CC)*+γ(CH)*
519vw				519vs	518vs	514vs	516sh	γ(CH)+δ(CC)*+γ(CH)*
508vw	510vw			508s	510s			γ(CH)
				476sh	481w	477w		δ(OSiC)
				465w			465w	δ(OSiC)+δ(CC)*+δ(CO)*
				456sh	453w	446vw		δ(OSiC)
				451w				δ(OSiC)
				435w	435w	432vw		δ(OSiC)
				425w				δ(OSiC)
				414w	413w	419vw		δ(OSiC)
	376vw	376vw						γ(CC)
283w	281vw	288vw	286vw					β(CC)+δ(CC)*+δ(CO)*+T'(Ln^{3+})
268w	267vw	269vw						T'(Ln^{3+})+lm
	257vw	257w						T'(Ln^{3+})+lm
239sh	237sh	237m						lm
231m	232m	230sh						lm
186sh	183sh	181sh						γ(CC)+lm
170m	170m	168m						lm
		151w						lm
109m	108m	119sh						lm
		109m						lm

[a]Key: vs, very strong; s, strong; m, medium; w, weak; vw, very weak;sh, shoulder; b, broad ν, stretching; ν_s, symmetric stretching; ν_{as}, antisymmetric stretching; δ, bending; γ, out of plane bending; β, in plane bending; ov/cb, overtone and/or combinational band; lm, lattice modes

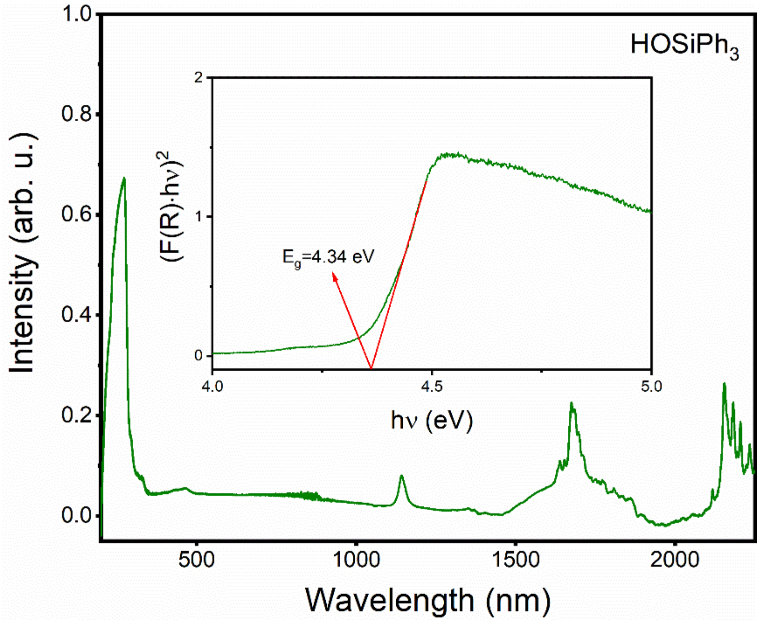

Figure S1. The absorption spectrum of the Ph_3SiOH ligand and the result of the energy band gap (Eg) calculation using the Kubelka-Munk theory.

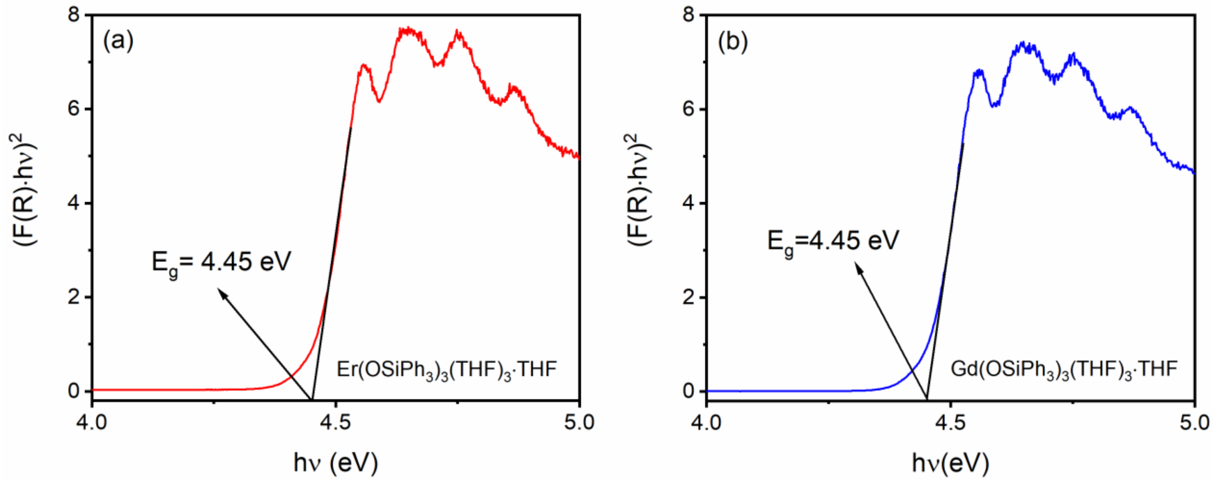

Figure S2. The results of the energy band gap (Eg) calculation using the Kubelka-Munk theory for the $Er(OSiPh_3)_3(THF)_3{\cdot}THF$ (a) and $Gd(OSiPh_3)_3(THF)_3{\cdot}THF$ (b) crystals.
